# PReS-FINAL-2204: Developing of a new scale for assessing the adherence to colchicines treatment in pediatric patients with FMF

**DOI:** 10.1186/1546-0096-11-S2-P194

**Published:** 2013-12-05

**Authors:** S Yesilkaya, C Acıkel, B Eren Fidanci, B Sozeri, N Aktay Ayaz, N Akıncı, G Ozcelik, S Kavukcu, Ü Aydogan, S Ozenc, S Emre, O Donmez, S Yuksel, A Delibas, A Berdelli, H Poyrazoglu, M Saldir, N Cakar, H Peru, S Bakkaloglu, Y Tabel, O Sari, A Polat, G Basbozkurt, E Unsal, F Gok, O Kasapcopur, S Ozen, E Demirkaya

**Affiliations:** 1 Gulhane Military Medical Academy, Ankara, Turkey; 2 FAVOR, Ankara, Turkey; 3 GATA Family Medicine, Ankara, Turkey; 4 Hacettepe University, Ankara, Turkey

## Introduction

Familial Mediterranean Fever (FMF) is a disease characterized by attacks and colchicine is the medication considered most effective in reducing the intensity and frequency of attacks. Adherence to the medication regiment is important not only to manage FMF symptoms, but also to prevent amyloidosis.

## Objectives

In this study, it is aimed to develop and assess the validity and reliability of the adherence scale for colchicine treatment in pediatric FMF patients.

## Methods

This study was planned as a methodological study to development of scale for assessment of adherence to treatment of pediatric patients with FMF using colchicine treatment. Pediatric patients (2-18 ages) with FMF using colchicine at least 6 months and accepted to participate in the study constitute the sample of the study. "Data collection forms about the sociodemographic and medical information (demographic, clinical and laboratory findings) of patients", "adherence scale for colchicine in pediatric FMF patients" and "Morisky Medication Adherence Scale" were used as data collection instruments. If the patient was under 7 years old, his parents filled the forms.

## Results

There were 150 patients with FMF enrolled for the validation of the study. The median age of the patients was 11.11 ± 4.02 (min.2.74-max.17.99) and 48.7% of them were male. The median of the attack frequency was 11,00 ± 10,74(min. 0-max 52) and 60.7% of the participants had irregular attacks.

For internal consistency, Cronbach's alpha was 0,728 for "adherence scale for colchicine in pediatric FMF patients". Also, there was a positive and significant correlation (r: 0.843, p < 0.001) between test and retest score. As a result it is accepted that this scale can be used for pediatric patients with FMF who use colchicine as a "reliable scale". As a "content validity" 13 experts' opinion were taken. As a "surface validity" a preliminary test was applied with 15 patients. For the "criterion validity" the correlation between this scale and Morisky medication adherence scale was evaluated and for the "structure" validity, factor analyzes and Kaiser-Meyer-Olkin tests were performed. According to the results this scale was accepted as a "valid" to assessing of adherence to treatment of pediatric patients with FMF who use colchicine.

## Conclusion

Based on these results, using this scale for the purpose of the assessment and follow up of adherence to treatment of pediatric patients with FMF who use colchicine is recommended.

## Disclosure of interest

None declared.

